# An approach to addressing subpopulation considerations in systematic reviews: the experience of reviewers supporting the U.S. Preventive Services Task Force

**DOI:** 10.1186/s13643-017-0437-3

**Published:** 2017-03-02

**Authors:** Evelyn P. Whitlock, Michelle Eder, Jamie H. Thompson, Daniel E. Jonas, Corinne V. Evans, Janelle M. Guirguis-Blake, Jennifer S. Lin

**Affiliations:** 1 Patient-Centered Outcomes Research Institute, 1919 M Street NW 2nd Floor, Washington DC, 20036 USA; 2 Kaiser Permanente Research Affiliates Evidence-based Practice Center, Center for Health Research, Kaiser Permanente Northwest, 3800 N. Interstate Ave, Portland, OR 97227 USA; 3 Department of Medicine, University of North Carolina Chapel Hill, 5034 Old Clinic Building, Chapel Hill, NC 27599 USA; 4 Department of Family Medicine, Tacoma Family Medicine Residency Program, University of Washington, 521 Martin Luther King Jr. Way, Tacoma, WA 98405 USA

**Keywords:** Patient subpopulation, Subgroup, Heterogeneity, Systematic review

## Abstract

**Background:**

Guideline developers and other users of systematic reviews need information about whether a medical or preventive intervention is likely to benefit or harm some patients more (or less) than the average in order to make clinical practice recommendations tailored to these populations. However, guidance is lacking on how to include patient subpopulation considerations into the systematic reviews upon which guidelines are often based. In this article, we describe methods developed to consistently consider the evidence for relevant subpopulations in systematic reviews conducted to support primary care clinical preventive service recommendations made by the U.S. Preventive Services Task Force (USPSTF).

**Proposed approach:**

Our approach is grounded in our experience conducting systematic reviews for the USPSTF and informed by a review of existing guidance on subgroup analysis and subpopulation issues. We developed and refined our approach based on feedback from the Subpopulation Workgroup of the USPSTF and pilot testing on reviews being conducted for the USPSTF. This paper provides processes and tools for incorporating evidence-based identification of important sources of potential heterogeneity of intervention effects into all phases of systematic reviews. Key components of our proposed approach include targeted literature searches and key informant interviews to identify the most important subpopulations a priori during topic scoping, a framework for assessing the credibility of subgroup analyses reported in studies, and structured investigation of sources of heterogeneity of intervention effects.

**Conclusions:**

Further testing and evaluation are necessary to refine this proposed approach and demonstrate its utility to the producers and users of systematic reviews beyond the context of the USPSTF. Gaps in the evidence on important subpopulations identified by routinely applying this process in systematic reviews will also inform future research needs.

## Background

The growing focus on patient-centered outcomes in health care has been accompanied by increasing interest in targeted, individualized recommendations for clinical care, including screening, preventive interventions, and treatment. This is driven in large part by the rising recognition that medical interventions do not affect all patients in the same way, a situation referred to as heterogeneity of intervention (treatment) effects [1]. Guideline developers and other users of systematic reviews seek information about whether a preventive or medical intervention is likely to benefit some patients more (or less) than the average and to understand which patients are at greatest (or least) risk of intervention-related harm [2]. As such, there has been a need to develop methods for dealing with heterogeneity of intervention effects to help address concerns about the inappropriate clinical application of average effects and to aid guideline developers in making recommendations tailored to specific subpopulations of patients when appropriate. Two recent surveys of existing practices and published guidance for considering clinical heterogeneity in systematic reviews found that there is little consensus and limited clear guidance to support consistent approaches to this important issue, although these are very much needed [2, 3].

To this end, we have developed and piloted a process for including patient subpopulation considerations into all phases of systematic reviews with two explicit goals: (1) to provide a consistent, systematic assessment of the evidence base for specific subpopulations within a given systematic review and (2) to provide the U.S. Preventive Services Task Force (USPSTF) with the information necessary to inform judgments about the appropriateness of general population versus subpopulation-specific clinical practice recommendations. The approach described in this paper was developed to provide practical guidance for the consistent application of subpopulation considerations in systematic reviews conducted by Evidence-based Practice Centers (EPCs) funded by the Agency for Healthcare Research and Quality (AHRQ) to support primary care clinical preventive service recommendations made by the USPSTF.

Background work conducted for this project included an examination of available information on how major guideline developers and groups setting standards for systematic reviews address subgroup analyses and subpopulation issues. We chose ten groups with particular relevance to primary care preventive services in the USA or internationally recognized for their well-developed methods (see Table 1). In November 2015, we reviewed their websites (and manuals or written procedures, where available) for descriptions of methods used to evaluate subgroup-specific evidence and address subpopulation issues.

**Table 1:** Summary of subgroup-specific information addressed by select guideline developers and groups setting standards for systematic reviews

	PHASE I Topic scoping and work plan development	PHASE II Data abstraction and critical appraisal	PHASE III Data analysis and synthesis	PHASE IV Reporting and interpretation
AAFP [42]				
AAP [43]				
ACOG [44]				
ACP [45, 46]				
CTFPHC [12]	✓	✓		
CPSTF [47, 48]				
Cochrane [7, 11, 49]	✓		✓	✓
GRADE [8–10, 20]	✓	✓	✓	✓
IOM [14, 50]	✓			✓
NICE [13]	✓			

*AAFP* American Academy of Family Physicians, *AAP* American Academy of Pediatrics, *ACOG* American Congress of Obstetricians and Gynecologists, *ACP* American College of Physicians, *CTFPHC* Canadian Task Force on Preventive Health Care, *CPSTF* Community Preventive Services Task Force, *Cochrane* The Cochrane Collaboration, *GRADE* Grading of Recommendations Assessment, Development and Evaluation Working Group, *IOM* Institute of Medicine, *NICE* National Institute for Health and Care Excellence

Based on our 15 years’ experience conducting systematic reviews for the USPSTF, and informed by relevant literature discussing subgroup analysis and subpopulation issues, we developed tools and methods for incorporation of subpopulation considerations into each of four phases of the systematic review process: (I) topic scoping and work plan (protocol) development, (II) data abstraction and critical appraisal, (III) data analysis and synthesis, and (IV) reporting and interpretation. We presented these tools and processes to the Subpopulation Workgroup of the USPSTF and revised our draft approach based on feedback from workgroup members. We refined our proposed methods based on pilot testing the approach on three reviews conducted for the USPSTF: *Aspirin for the Primary Prevention of Cardiovascular Events*, *Screening for Lipid Disorders in Adults*, and *Screening for Obstructive Sleep Apnea in Adults* [4–6].

## Main text

### Key concepts and definitions

We use the terms “subgroup” and “subpopulation” to refer to distinct elements, such that “subpopulations” refer to groups of individuals that are the target of policy or practice recommendations, and “subgroups” refer to specific types of analyses undertaken on a subset of participants (see Table 2). In the context of systematic reviews, differences between studies (i.e., heterogeneity) must be considered to appropriately summarize a body of evidence, including making decisions about whether or not to quantitatively combine results [7]. Heterogeneity considerations should inform decisions made about the review scope and methods during protocol development, including planned approaches to data abstraction and data synthesis, as well as final interpretation of review findings. The set of included studies for a systematic review question can differ somewhat or substantially in dimensions underlying heterogeneity: the populations studied, the interventions investigated, and the outcomes measured, as well as in the methods underpinning each study’s findings. These differences can be understood as clinical, methodological, and statistical heterogeneity, all of which inform the synthesis of evidence within a systematic review (see Table 2) [7].

**Table 2:** Definitions of terminology used

Term	Description/definition	Example(s)
Subgroup	The term “subgroup” describes an analysis of a subset of participants (e.g., selected set of individuals with specific patient characteristics within an individual study or across studies in the case of individual patient data meta-analyses).	“Subgroup analyses are often performed to identify characteristics within the study population that are associated with greater benefit from the intervention, with no benefit, or even with harm” [37].
Subpopulation	The term “subpopulation” describes a specific group of individuals with common patient characteristics (e.g., race/ethnicity, age, risk factors) that is the target of an intervention or a policy recommendation.	“If a subpopulation may not benefit from the therapy, it is important to identify the subpopulation and verify this finding in an appropriate clinical trial” [37].
Clinical heterogeneity	Variability between studies in the populations enrolled, the active interventions and comparison interventions they receive, or selection and timing of measured outcomes [3, 7].	Patient characteristics • Socio-demographics • Baseline risk Study characteristics • Intervention • Comparators • Outcome measurement
Methodological heterogeneity	Variability in study design and conduct that can lead to differences in measured intervention effects due to non-comparability or bias [3, 7].	Risk of bias • Study design • Study conduct • Study analysis
Statistical heterogeneity	Measured variability in observed intervention effects between studies that are greater than would be expected due to chance (random error) [3, 7].	Statistical tests • *I* ^2^ • Cochran’s Q test
Within study	The term “within study” refers to the framework in which comparisons or analyses are conducted; in this case, researchers are examining the variation or impact of factors (e.g., populations, interventions, outcomes) within one study or trial.	"In single trials, the comparison [between subgroups] is always *within studies*: that is, the two groups of patients (e.g., the older and younger) or the two alternative ways of administering the intervention (e.g., higher and lower doses) were assessed in the same RCT" [26].
Between study	The term “between study” refers to the framework in which comparisons or analyses are conducted; in this case, researchers are examining the variation or impact of factors (e.g., populations, interventions, outcomes) across multiple studies or trials.	"The inference regarding the effect is, however, limited because this was a *between study* rather than a within study comparison. As a result there are a number of competing explanations for the observed differences between the high- and low-dose studies” [26].
Study level	The term “study level” is used to describe the unit of inquiry or data source being considered by systematic reviewers; in this case, data from a single study or trial are evaluated.	"The ideal way to study causes of true variation is within rather than between studies. In most situations however, we will have to make do with a *study level* investigation \and hence need to be careful about adjusting for potential confounding by artefactual factors such as study design features" [51].
Body of evidence level	The term “body of evidence level” is used to describe the unit of inquiry or data source being considered by systematic reviewers; in this case, data from a group of studies are evaluated.	"Systematic review and guideline authors use this [GRADE] approach to rate the quality of evidence for each outcome across studies (i.e., for a *body of evidence*)" [52].

Clinical heterogeneity reflects variation between studies in the populations enrolled, in the active interventions and comparison interventions they receive, or in selection and timing of measured outcomes [2]. When there are variable intervention effects across studies, investigating how these differences may be related to effect variation can inform targeting or tailoring research information to specific populations, situations, or circumstances. Clinical heterogeneity is the type of heterogeneity most related to subgroup and subpopulation issues and therefore of major interest to clinical and policy-level decision-makers [3]. Methodological heterogeneity reflects differences in study design and conduct, including risk of bias, across studies in the systematic review [7]. Methodological heterogeneity can lead to differences in measured intervention effects, but these reflect artifacts of the research process rather than clinically relevant differences. Finally, statistical heterogeneity is revealed through statistical testing as to whether measured differences in intervention outcomes between studies in the body of evidence are greater than would be expected due to chance (generally *p* < 0.05) [2]. The job of the systematic reviewer is to understand the interrelationships of these factors, to control for (or investigate) them in assembling and analyzing a body of evidence to answer a particular question, and to summarize and communicate their implications for decision-makers.

### Existing guidance

Both guideline developers and systematic reviewers working on behalf of guideline developers have a strong interest in specifying credible, relevant methods for fairly and consistently considering subgroup findings from primary studies and subpopulation differences in intervention effects. To inform our work, we therefore examined how selected prominent guideline developers or groups setting standards for systematic reviews address these two issues. Table 1 summarizes the subgroup-specific information addressed by selected guideline and review groups for each phase of a systematic review. Our review revealed that prominent guideline developers typically lack detailed information about how to plan and use subgroup analyses. While some of the review or guideline groups addressed the issue of handling subgroup data conceptually or in detail for a specific aspect of the systematic review, no group outlined a comprehensive approach to integrating subgroup considerations and analyses into all phases of the systematic review process.

GRADE provided the most comprehensive guidance on inclusion of subgroups in all phases of systematic reviews and is the only group that addressed credibility assessment of subgroup analyses [8–10]. The Cochrane Collaboration Handbook also included guidance on the use of subgroup analyses in reviews and addressed a priori selection of a small number of study characteristics for subgroup analyses that are supported by scientific evidence, how to analyze subgroup data to investigate heterogeneity, and interpretation of subgroup analyses, including caveats such as the potential for bias since subgroup comparisons are not usually accounted for by the randomization approach [3, 7]. In their description of methods for child health reviews, the Cochrane Child Health team also provides questions regarding age-based treatment effects to guide the planning of a priori subgroup analyses [11].

Selected other groups included in our scan (i.e., the Institute of Medicine (IOM), the National Institute for Health and Care Excellence (NICE), the Community Preventive Services Task Force (CPSTF), and the Canadian Task Force on Preventive Health Care (CTFPHC)) touched briefly on subgroup considerations for one or two of the systematic review phases. Description of subgroup methods during review scoping and work plan development was generally limited to a series of questions to guide the inclusion of subgroups [12, 13] or to specifying the standard that reviewers should describe and justify a priori any planned subgroup analyses, in the case of the IOM [14]. Information from these groups on reporting and interpretation of subgroup findings consisted of a few elements that should be reported by reviewers (e.g., clinical and methodological characteristics of studies) [7, 14]. Related efforts on equity-focused reviews and clinical guidelines by the PRISMA-Equity Bellagio group [15] and NICE [16], respectively, highlight the importance of addressing health disparities in systematic reviews. The PRISMA-Equity Bellagio group focuses on improved reporting in equity-focused systematic reviews, including items such as presentation of subgroup analyses [15]. The NICE guideline on addressing equality issues includes scoping discussions about inequalities in prevalence, risk factors, or severity and a priori identification of relevant subpopulations [16].

The professional society websites we reviewed (i.e., American Academy of Family Physicians (AAFP), American Academy of Pediatrics (AAP), American Congress of Obstetricians and Gynecologists (ACOG), American College of Physicians (ACP)) did not include any information about how subpopulations are considered in their guidelines or address how subgroup considerations are incorporated in the reviews of the evidence on which their guidelines are based.

### Proposed approach

**Fig. 1:**
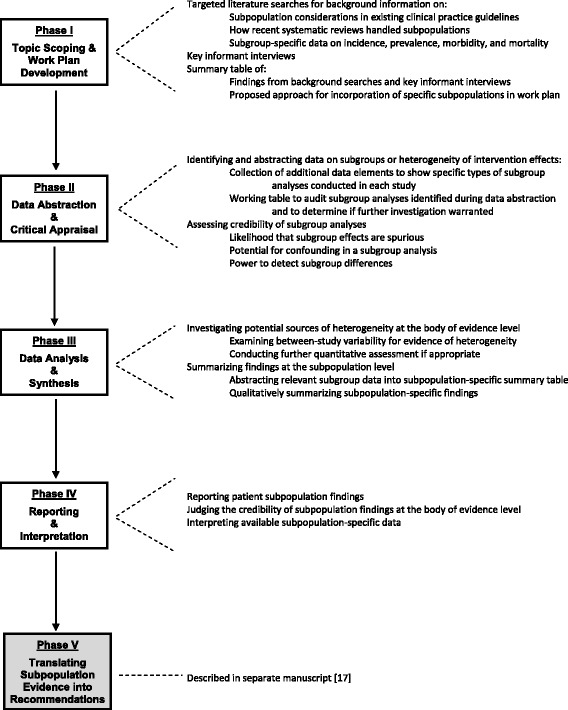
Major phases of systematic reviews and corresponding subpopulation processes

Below we describe the methods we developed for incorporating subpopulation considerations into the four major phases of a systematic review: (I) topic scoping and work plan (protocol) development, (II) data abstraction and critical appraisal, (III) data analysis and synthesis, and (IV) reporting and interpretation (Fig. 1). We developed these approaches primarily to support our work conducting systematic reviews for the USPSTF given their need to judge the appropriateness of general population versus subpopulation-specific clinical practice recommendations. The process of translating the subpopulation evidence presented in systematic reviews into clinical practice recommendations is described in another manuscript [17]. Below we provide examples for many of our processes and tools based on our systematic review experiences with the USPSTF.

### Phase I: topic scoping and work plan development

#### Topic scoping

Decisions about which subpopulations will be investigated in a systematic review should be based on understanding of the existing evidence base [18]; therefore, the first step in exploration of important subpopulations during topic scoping involves targeted literature searches informed by clinical consultation as necessary. These literature searches include:

- How other guideline groups have recently handled subpopulation considerations for the topic
- How other recent, well-conducted systematic reviews have handled subpopulation considerations for the topic
- Data on incidence, prevalence, morbidity, and mortality for the condition of interest by age, sex, race/ethnicity, and important topic-specific clinical characteristics

The collected information is used to identify presumptive subpopulations of interest, understand the issues within the literature related to relevant subpopulations, and develop a set of questions for key informant interviews consisting of two to four clinical and content experts in the systematic review topic area. Key informant candidates may include, for example, previous reviewers for the specific content area, authors of validated risk assessment tools, principal investigators of large trials that include subgroup analyses, leaders in professional societies relevant to the clinical topic, or members of clinical guideline panels. The purpose of conducting key informant interviews is to learn which subpopulations experts would be most concerned about being given a general population screening and/or treatment recommendation, as opposed to a subpopulation-specific recommendation, and why.

Key informants can help determine what is known about sources of heterogeneity of intervention effects (e.g., prior subgroup analyses, dose-response relationships, or differences in outcomes) and known or concern about potential subpopulation differences for the topic. Candidate patient-level variables to define subpopulations include age, sex, race, ethnicity, comorbidities, baseline disease risk, disease severity or other important disease features, genetic variants, or psychosocial variables with a clear scientific rationale as a treatment effect modifier [19]. Key informant questions can confirm or query important issues on potential mechanisms of preventive services heterogeneity within specific subpopulations (e.g., differing baseline risk of disease-related outcomes, competing risks/limited life expectancy, varying risk(s) of intervention harm(s), variable responsiveness to the preventive intervention, differential impact of time to benefit or to harm, primary and differing values for patient-important outcomes). Table 3 shows how these mechanisms might affect questions about heterogeneity for different types of clinical preventive services to support development of questions for key informants. Experts can help identify epidemiological data to support potential mechanisms of heterogeneity as well as validated risk assessment tools or large multivariable analyses showing the combined impact of potential subgroup factors on outcomes for the condition of interest. Table 4 provides sample questions to guide reviewers in developing topic-specific questions for obtaining feedback from key informants.

**Table 3:** Potential drivers of heterogeneity of intervention effects for different types of clinical preventive services

	Potential drivers of heterogeneity of intervention effects				
Screening	Baseline risk	Vulnerability/risk of harms	Variable responsiveness to preventive intervention		Specific primary and patient-important outcomes
A. When might screening for disease risk factors result in differing net benefits for subpopulations defined by age^a^, race-ethnicity, sex, or other targeting factors?					
Screening test for risk factors	Does [the targeting factor] modify the prognostic significance of risk factors or change their prevalence (e.g., does family history carry the same relative risk in older individuals as in younger)? Does baseline risk for the disease outcome of interest directly vary by [the targeting factor] (e.g., risk of hypertension increases with age)?	Does screening involve invasive, complicated screening tests, and [the targeting factor] that might influence risk of screening harms or willingness to be screened?	Do risk factors for the disease outcome of interest for this screening test vary by differences in [the targeting factor]? Does presence of the [targeting factor] change approaches to risk factor detection (e.g., fat redistribution with age)?		Would patient-important outcomes or values about this test differ substantially in the subpopulation defined by [the targeting factor]?
Risk factor modification	Does [the targeting factor] modify the relative benefits associated with risk factor reduction (e.g., is the same degree of weight reduction in men and women associated with the same health benefits)?	Does [the targeting factor] increase the vulnerability to harms from risk factor modification (e.g., caloric restriction may induce malnutrition)?	Does [the targeting factor] modify the responsiveness to interventions for risk factor reduction (e.g., caloric restriction may not induce weight loss due to lower resting metabolism with older age)?		Would values about this intervention or about the patient-important outcomes resulting from this risk factor intervention differ substantially in the subpopulation defined by [the targeting factor]?
B. When might screening for diminished function result in differing net benefits for subpopulations defined by age^a^, race-ethnicity, sex, or other targeting factors?					
Screening test for reduced function	Does risk of reduced function vary substantially by [the targeting factor]?	Does screening involve invasive, complicated screening tests, and [the targeting factor] that might influence risk of screening harms or willingness to be screened?	Does screening for reduced function vary in effectiveness or by optimal approach, depending on [the targeting factor] (e.g., is thyroid stimulating hormone equally effective in screening for hypothyroidism in all subpopulations)?		Would patient-important outcomes or values about this test differ substantially in the subpopulation defined by [the targeting factor]?
Usual treatment to restore function or ameliorate dysfunction	Does risk of functional impairment without treatment vary by [the targeting factor] (e.g., different natural history)?	Do potential harms of usual treatment increase due to vulnerabilities associated with [the targeting factor] (e.g., greater falls with vision correction in older adults)?	Is there variable responsiveness to interventions to improved function among those defined by [the targeting factor] that would further support the value of early intervention (e.g., visual correction also affecting cognitive development in young children)?		Would values about this functional intervention or about the patient-important outcomes resulting from this treatment to restore function differ substantially in the subpopulation defined by [the targeting factor]?
C. When might screening for potentially fatal or disabling conditions result in differing net benefits for subpopulations defined by age^a^, race-ethnicity, sex, or other targeting factors?					
Screening test for fatal or disabling conditions	Does risk of disease vary substantially by [the targeting factor]? Does natural history vary substantially by [the targeting factor]?	Does screening involve invasive, complicated screening tests that might influence risk of screening harms or willingness to be screened in those with [the targeting factor]? Does risk of overdiagnosis vary by [the targeting factor]?	Does screening for potentially fatal or disabling conditions vary in effectiveness or by optimal approach, depending on [the targeting factor]?		Would patient-important outcomes or values about this test differ substantially in the subpopulation defined by [the targeting factor]? Is there heterogeneity in the components of a composite outcome by [the targeting factor]?
Treatment of screen-detected disease	Does screen-detected disease generally have different prognosis by [the targeting factor]?	How do treatments with potential harms, med-med interactions, or comorbidity interactions affect vulnerability/risk of harms by [the targeting factor]?	Is there variable responsiveness to preventive intervention if disease detection differs by [the targeting factor]?		Would values about this disease treatment or about the patient-important outcomes resulting from this disease treatment differ substantially in the subpopulation defined by [the targeting factor]?
Chemoprevention		Potential drivers of heterogeneity of intervention effects			
		Variable responsiveness to preventive intervention	Vulnerability/risk of harms	Variable responsiveness to preventive intervention	Specific primary and patient-important outcomes
D. When might chronic or acute disease chemopreventive interventions result in differing net benefits for subpopulations defined by age^a^, race-ethnicity, sex, or other targeting factors?					
Chemoprevention of chronic disease	Identify candidates for chemopreventive medication	Does [the targeting factor] affect baseline risk for one or more outcomes of interest and help define candidates for chemoprevention (e.g., age and risk of gastrointestinal bleeding with aspirin use)? Is [the targeting factor] part of formal risk assessment tools to identify medication candidates?	Does [the targeting factor] interact with other factors (e.g., medical history, comorbidities, cotreatments) to modify the eligibility for chemoprevention?	Do chemopreventive medications require certain supports and systems that are variously available to subpopulations defined by [the targeting factor] in order to be effective?	Does the disutility associated with the requirements for chemoprevention vary by [the targeting factor]? Are there other barriers or facilitators that vary by [the targeting factor]?
	Deliver chemopreventive medication	Is [the targeting factor] an independent risk factor for the outcomes to be prevented, thereby increasing potential absolute benefit?	Does [the targeting factor] directly increase the risk of chemoprevention-related adverse effects, or indirectly, through a greater likelihood of treatment risk modifiers, such as comorbidities, or cotreatments?	Is [the targeting factor] associated with loss of/difference in responsiveness to mechanisms of prevention? Is [the targeting factor] associated with important differences in compliance needed for benefit (e.g., dementia)?	Would patient-important outcomes or values about chemopreventive medications differ substantially in the subpopulation defined by [the targeting factor]?
Acute disease chemoprevention	Identify candidates for chemopreventive medication	Is there a higher baseline risk of infectious disease diagnosis or sequelae [the targeting factor]?	Does [the targeting factor] interact with other factors (e.g., medical history, comorbidities, cotreatments) to modify the eligibility for chemoprevention?	Do chemopreventive medications require certain supports and systems that vary in groups defined by the targeting factor] in order to be effective?	Are there barriers or facilitators for taking chemopreventive medication that vary by [the targeting factor]?
	Deliver chemopreventive medication	Is there a larger absolute benefit by [the targeting factor] due to higher risk of disease-related outcomes?	Does risk of harms with the chemopreventive medication vary in those defined by [the targeting factor]?	Is [the targeting factor] associated with loss of/difference in responsiveness to mechanisms of prevention? Is [the targeting factor] associated with important differences in compliance needed for benefit (e.g., dementia)?	Would patient-important outcomes or values about chemopreventive medications differ substantially in the subpopulation defined by [the targeting factor]?
Intervention		Potential drivers of heterogeneity of intervention effects			
		Baseline risk	Vulnerability/risk of harms	Variable responsiveness to preventive intervention	Specific primary and patient-important outcomes
E. When might complex interventions for potentially fatal or disabling conditions result in differing net benefits for subpopulations defined by age^a^, race-ethnicity, sex, or other targeting factors?					
Identify complex intervention candidates	Is [the targeting factor] a marker for selected deleterious health events that are amenable to complex interventions?	Is [the targeting factor] also a marker for potential harms from complex interventions?	Do complex interventions require certain supports and systems that vary by [the targeting factor] in order to be effective?	Are there barriers or facilitators of complex interventions that vary by [the targeting factor]?	
Complex or behavioral intervention delivery	Is [the targeting factor] associated with increased baseline risk of some adverse health events (e.g., falls prevention, suicide, fatal motor vehicle accidents if age is the targeting factor)?	Most interventions have few harms other than opportunity costs. Are there any harms related to [the targeting factor] that may not be hypothesized (e.g., increased visual acuity correction as an age-related harm)?	Do certain conditions (e.g., dementia), decreased function, or inadequate environmental support affect intervention effectiveness?	Would patient-important outcomes from or values about complex or behavioral interventions differ substantially in the subpopulation defined by [the targeting factor]?	

^a^Extremes of older age are often a proxy for reduced life expectancy, although multiple comorbidities may also be a marker for this state. Reduced life expectancy can be an additional subpopulation factor modifying potential net benefit for preventive topics in which time to benefit is prolonged, particularly when the harms are likely in a shorter time frame.

**Table 4:** Key informant interview sample questions

Are there important advances in research or clinical thinking since [insert year of previous review] that would suggest looking at the same (e.g., age, sex, risk-defined) and/or other specific subpopulations (e.g., race/ethnicity, co-morbidities, co-interventions)? Which subpopulations are most important? What streams of evidence since [insert year of previous review] support your perspective? Are there key studies we should be aware of in formulating our approach to subpopulations?
*Greater benefits from screening can occur in those who are more likely to be undiagnosed, and from intervention in those at higher risk.* Does under-diagnosis vary by age, sex, race/ethnicity or other characteristics? Does absolute risk vary by age, sex, race/ethnicity or other characteristics? For which subpopulation(s) would benefit from screening and intervention be substantially greater than “average”? Why?
*Lesser benefits from screening and intervention can occur in those with competing risks, health states, or limited life expectancy, which reduce the likelihood of benefit from successful intervention or affect the ability to accurately screen for this condition.* Are there subpopulations that might be substantially less likely to benefit from detection and intervention? Why?
Do the values that patients place on important outcomes (benefits or harms) associated with this topic differ by age, sex, race/ethnicity or other characteristics? Please be specific. Based on your answers to these questions, which subpopulations differ substantially enough in the likelihood of benefits (and/or risk of harms) from screening and intervention of [insert topic] that they may warrant different clinical preventive recommendations? What criteria would you use to define these clinically relevant subpopulations? Should this topic be scoped to specifically include a high-risk approach in addition to (or instead of) a general population approach? What are the validated risk assessment tools that are applicable to this topic? Are some of the tools better than others for framing a potential high-risk approach to [insert topic]? Do any tools vary in their applicability to specific subpopulations based on age, sex, race/ethnicity, comorbidities, or other factors? Is the epidemiological information below [paste data below this question] that we have located to frame this topic complete, current, and representative of the issues for subpopulations in [insert topic] (i.e., Do the data adequately capture the extent to which death or morbidity from [insert condition(s)] differ by age, sex, race/ethnicity, or other clinical characteristics?)? Are there other data sources we should use to frame this topic?

In our experiences with implementing this approach, we confirmed the value of eliciting expert input into our subpopulation considerations early in the review process. These experts can often provide guidance about important resources (e.g., presentations from relevant professional society meetings) or ongoing research that would otherwise take considerable effort to locate. By efficiently helping us understand the perspectives of clinical and research experts, we could more quickly focus on subpopulations with sufficient prior evidence or controversy to guide our protocol development. We also found that a conference call format (conducted one-on-one or with a few individuals) may be more conducive to gathering detailed expert perspectives with accompanying rationale and allows for easy clarification of complex statements. Eliciting expert feedback via email, however, can still provide valuable information with limited time and effort expended.

### Work plan development

Work plan development for a systematic review includes drafting an analytic framework, research questions, and inclusion/exclusion criteria that specify the logic and scope of the review, including the populations, interventions, comparators, and outcomes of interest. An analytic framework is a graphic representation of linkages between interventions and outcomes that helps to identify the questions that the review is addressing [7, 20–22]. The background searches and key informant interviews described above help determine whether and how relevant subpopulations will be incorporated into the analytic framework, research questions, and inclusion/exclusion criteria that guide the literature searches, data abstraction, and analysis processes in later phases of the systematic review.

We developed a summary table to assist reviewers in presenting the findings from the topic scoping process, including the key informant interviews, and outlining recommendations for incorporation of specific subpopulations into the work plan for consideration and approval by AHRQ and the USPSTF. Table 5 provides an example of a completed summary table for a review on aspirin for the primary prevention of cardiovascular events [5]. The primary purpose of the table was to support the a priori selection of a limited number of patient subpopulations to be examined in the systematic review and to provide the rationale for inclusion of these subpopulations. The six columns in the table are defined as:

**Table 5:** Summary of work plan guidance for subpopulation considerations—*example*. Aspirin for prevention of cardiovascular events

Potential subpopulation	Applicable to review updates only		Applicable to new reviews and review updates			
	(A) Previous systematic review’s approach for this subpopulation	(B) Previous separate subpopulation recommendation statement?	(C) Importance of a priori designation	(D) Rationale for importance determination for this review	(E) Policy context	(F) Proposed work plan approach
Age	- Age not explicitly addressed in key questions - Reported results of age-specific subgroup analyses from primary papers - Recommendation statement cites substantial evidence for differential benefits by age in the form of risk assessment tables	Yes	High	Increasing potential benefit for aspirin as people get older due to increased baseline risk for cardiovascular disease (CVD); this is balanced against increasing potential harm as people get older and experience increased risk for gastrointestinal bleeding.	- Addressed in recent meta-analyses - Age is a principal component of CVD risk and risk assessment; there is wide availability of validated risk assessment tools including age in user-friendly formats	In 2009, the USPSTF recommended aspirin for men 45–79 and women 55–79 when the potential benefit of CVD event reduction outweighs the risk of gastrointestinal bleeding. There was insufficient evidence for adults 80 and older and a recommendation against aspirin for men younger than 45 and women younger than 55. Continue to address age-specific subgroups. Establish age as an a priori subgroup; gather, analyze, and report evidence by age-specific subgroups. Attend to age 80 and older for evidence sufficiency and future research
Sex	- Separate key questions for men and women for benefits and harms	Yes	Highest	Epidemiology of CVD events is different for men and women; men have a higher risk for events and have events at younger ages. Men are also at higher risk for gastrointestinal bleeding.	- Recently cited as most important subgroup by key informants - Controversial: recent meta-analyses including new trial data suggest no differences in the benefit of aspirin by sex, which is different from the previous review that found a significant benefit in women for stroke (but not myocardial infarction (MI)) and a significant benefit for men in MI (but not stroke)	Continue to explicitly address sex-specific subgroups. Establish sex as an a priori subgroup; gather, analyze, and report evidence by sex-specific subgroups.
Race/ethnicity	- Race/ethnicity not addressed in the previous review	No	Unknown due to lack of evidence Perceived need for information	Due to disparities in incidence and mortality of CVD, particularly among Blacks, there is the potential for greater benefit from aspirin in this group.	- Not addressed in recent meta-analyses - Key informant indicates a lack of evidence for this subgroup	Based on the recent work of others and key informant input, evidence reported by race/ethnicity is not expected. However, if any subgroup data is reported in the literature, it should be captured, analyzed, and reported. Confirmed lack of subpopulation data should be reflected in Future Research section of the report.
Other risk-related subgroups	- Not explicitly addressed in previous key questions - Results of subgroup analyses from primary papers reported for the following groups: • Diabetics • Baseline blood pressure levels • Smoking status • Kidney function	No	Moderate	Possible biological plausibility for subgroup differences. Factors related to diabetes (e.g., hyperglycemia, hyperinsulinemia, increased oxidative stress, advanced glycosylation end products) may influence platelet activity. Patients with peripheral artery disease (PAD) or diabetes may have less response to aspirin due to high inflammatory burden and platelet activation. Concomitant medications (e.g., statins, angiotensin-converting enzyme inhibitors, fibrates, selective serotonin re-uptake inhibitors) influence platelet activity and bleeding risk.	- 3 new RCTs since last review in higher risk populations (2/3 in diabetics and 2/3 in patients with PAD) - Key informant identified patients with PAD as a priority - Recent meta-analyses have addressed the following subgroup considerations: CVD risk, smoking, diabetes, and cholesterol and blood pressure - Recent meta-analyses in diabetic and elevated blood pressure patients - A public comment cited a subgroup analysis from the Women’s Health Study showing that aspirin use was associated with increased harm in current female smokers. Because smoking is associated with both increased cardiovascular risk and gastrointestinal complications, this reviewer called for a cautious approach to aspirin use in female smokers	Establish CVD risk groups a priori for consideration of benefits and harms, including diabetes, PAD, blood pressure, and smoking.

(A) *Previous systematic review’s approach:* How each subpopulation of interest was addressed in the previous systematic review, if at all.
(B) *Separate recommendation statement:* Whether the guidance included a separate recommendation statement for each subpopulation of interest.
(C) *Importance:* Initial summary rating of the importance of each subpopulation relative to others suggested for inclusion in the systematic review to inform parsimonious selection.
(D) *Rationale:* Summary of information that supports each subpopulation as important and relevant to the systematic review (e.g., epidemiological trends, biological plausibility), including how key informant input supports the rationale for each subpopulation.
(E) *Policy context:* How recent reviews, meta-analyses, and clinical practice guidelines address preventive services recommendations for each identified subpopulation, including any disagreement across guidelines and reviews and how key informant input supports the policy importance of each subpopulation.
(F) *Proposed work plan approach:* Whether each subpopulation is proposed to be one of the a priori subpopulations for this review, and potential approaches to including it in the work plan, including hypothesized direction of effect, impact on net benefit, and mechanisms of action, if known.

As a result of the application of this process, the review designated age and sex as the a priori subgroup analyses and subpopulations of highest importance for the systematic review to update evidence addressing both benefits and harms [5]. We listed other cardiovascular disease (CVD) risk factors (including smoking, diabetes, blood pressure, and peripheral artery disease (PAD)) as important to examine for potential effect modification in terms of aspirin’s benefits, in particular, and listed selected medications, including selective serotonin reuptake inhibitors and non-aspirin non-steroidal anti-inflammatory drugs, as modifiers of potential harms of treatment only. A focused, a priori approach can be criticized for not being comprehensive; however, it conforms to guidance for parsimonious selection of a priori subgroups [19] and is important for feasibility. It does not preclude exploratory findings, when noted as such, or limit the span of issues that can be addressed in future updates.

### Phase II: data abstraction and critical appraisal

#### Data abstraction

Data abstraction is one of the most important and time-consuming steps of a systematic review [7]. The data collection instrument (e.g., evidence table, database, web-based systematic review software) is designed to extract critical and relevant data from eligible studies, including the details of the study design and conduct, characteristics of the population, specific outcomes assessed at specific times, intervention details, types of comparators, and, when appropriate to the topic, baseline risk levels of the study population. These components may be further categorized and summarized during data analysis and synthesis to allow for investigation of variability in methodological or clinical factors (see phase III: data analysis and synthesis).

In order to capture specific types of subgroup analyses conducted in each study, reviewers can make note during routine data abstraction of which a priori subpopulations identified in the work plan had subgroup-specific analyses reported. For the purposes of tracking the types of subgroup data available in studies, reviewers may also make note of which other subpopulations not specified a priori in the work plan had subgroup analyses reported.

After initial data abstraction, a working table (Table 6) can be used to audit the availability of subgroup-specific analyses in the body of evidence to determine whether it is feasible or worthwhile to further investigate a priori subpopulations of interest. Results from the audit (Table 6—Column 5) provide the rationale for whether or not to pursue further investigation of subgroup analysis results and can later be reported in the methods section of the evidence synthesis report. Within the working table, it is helpful to track the number of studies reporting subgroup analyses for the subpopulation of interest out of the total number of included studies in the review. As warranted, relevant subpopulation-specific summary tables can be developed during data analysis and synthesis.

**Table 6:** Audit for decision support

Audit of subgroup analysis results				
(1) List all a priori subpopulations from work plan	(2) List all studies conducted in a subpopulation only (e.g., older adults, males, females, diabetics)	(3) List all studies that reported subgroup analyses for this subpopulation	(4) List all outcomes reported for each subgroup analysis for this subpopulation	(5) Summarize decisions regarding further investigation of subgroup analysis results
- Age		- Study A (*n*) - Study B (*n*) - Study C (*n*) - Study D (*n*)	Outcome #1 - Study A - Study B Outcome #2 - Study C	- The majority of the studies (*x*/*y*) in the review reported age-related subgroup analyses. Age-related subgroup results will be abstracted in a separate table.
- Sex	- Study X (men) (*n*) - Study Y (men) (*n*) - Study Z (women) (*n*)	- Study A (*n*) - Study B (*n*) - Study C (*n*)	Outcome #1 - Study A - Study B Outcome #2 - Study C	- The majority of the studies (*x*/*y*) in the review were either conducted in males or females only, or some type of sex-related subgroup analysis was conducted. Sex-related subgroup results will be abstracted in a separate table.

### Critical appraisal

Another essential step in systematic reviews is critical appraisal of the design and conduct of studies [7, 23]. In addition to rating the quality of individual studies, assessing the credibility of subgroup analyses reported in studies is necessary when addressing subpopulation considerations in a review [8]. Many subgroup-specific claims made in trial reports are not credible, and key criteria for credibility should be addressed by the study authors [24, 25]. These criteria consider type I errors (spurious findings due to chance or confounding) and type II errors (failure to detect effects due to power).

General study quality issues (e.g., differential attrition) may affect the interpretation of subgroup-specific findings. Similarly, issues that affect subgroup validity may impact overall ratings of study quality. Subgroup analyses from poor-quality studies are at high risk of bias regardless of the credibility of the subgroup analyses.

Systematic reviewers can assess the credibility of subgroup findings for a priori subpopulations using Tables 7 and 8. Reviewers may consider collecting the data necessary for evaluating the credibility of subgroup analyses (e.g., a priori specification of analyses, interaction testing) during data abstraction to obviate the need for another close reading of the article. Using Table 7, for each study, reviewers can enter a row for each a priori subpopulation that specifies whether a subgroup effect was detected (based on interaction testing or point estimates and confidence intervals) and provide assessments of three domains related to credibility: (1) the likelihood that positive subgroup effects are *spurious*, (2) the potential for *confounding* in a subgroup analysis by another study variable (relevant to positive or negative subgroup findings), and (3) whether a trial was *powered* to detect subgroup differences, which is primarily relevant to a finding of no subgroup differences.

**Table 7:** Credibility assessment of subgroup analyses

Study name	Sub-population	Was a subgroup effect detected?	Likelihood that subgroup effects are SPURIOUS	Likelihood of CONFOUNDING of subgroup analysis	Likelihood of inadequate POWER to detect subgroup differences	Overall rating^a^
Study A	Ex. Age	Indicate whether the study found a difference in effects for this subgroup (i.e., yes, no)	Enter credibility assessment here (e.g., very likely, somewhat likely, unlikely, unclear, not applicable) and any relevant notes	Enter credibility assessment here (e.g., very likely, somewhat likely, unlikely, unclear) and any relevant notes	Enter credibility assessment here (e.g., very likely, somewhat likely, unlikely, unclear) and any relevant notes	Enter overall credibility rating here (e.g., low, moderate, high, or uncertain) and any relevant notes, including overall quality concerns for the study

^a^The overall rating should reflect consideration of general quality issues in the study.

**Table 8:** Framework for assessing credibility of subgroup analyses

	Questions to consider for credibility assessment
Likelihood that subgroup effects are SPURIOUS	MAIN DOMAIN: Was a statistical test for interaction performed and did it indicate effect modification? [24, 53] The statistical test of subgroup-intervention effect interaction assesses whether the effect differs significantly between subgroups, rather than only assessing the significance of the intervention effect in one subgroup or the other [54]. If the *p* value for the test result is <0.05 (or a more stringent alpha), then the effects between subgroups are not the same [54]. If there are multiple subgroup-treatment effect interactions, further statistical analyses are required to confirm whether the effects are independent [54]. When was the subgroup-specific analysis specified? Determine when the subgroup analyses were specified in the study [24, 54]. An a priori subgroup analysis is one that is planned and documented before examination of data, preferably in the study protocol, and ideally includes a hypothesized direction of effect. When reported, this information can often be found in the methods section of the article. Subgroup treatment effect interactions identified post hoc must be interpreted with caution. There are no statistical tests of significance that are considered reliable in this scenario [54]. Was the total number of subgroup analyses limited to a small number of clinically important questions (i.e., <5)? This is a *study*-specific factor, rather than a *subgroup*-specific one. Subgroup analyses should be limited to a small number of clinically important questions in each study, and ideally limited to the primary trial outcome [8, 54]. Sun et al. suggest there should be five or fewer subgroup hypotheses tested [24]. If conducting a large number of subgroup analyses, was the statistical significance threshold adjusted (e.g., using a lower *p* value than 0.05)? This is a *study-*specific factor. Because the probability of a false positive result is high when a large number of subgroup analyses are conducted, studies can correct for the inflated false positive rate by adjusting the significance threshold for their interaction tests [55]. For example, if 10 tests are conducted, each one could use a 0.005 threshold; if 20 are conducted, each one could use a 0.0025 (these thresholds were calculated using 0.05/*K*, where *K* is the number of independent tests conducted; this equation ensures that the overall chances of a false positive result are no greater than 5%) [55].
Likelihood of CONFOUNDING of subgroup analysis	MAIN DOMAIN: Was the subgroup analysis potentially confounded by another study variable? In subgroup analyses in RCTs, the primary intervention is randomized but the secondary factors defining subgroups usually are not [56]. Controlling for confounding variables for the secondary factor that defines a particular subgroup is important when investigators are interested in intervening using the subgroup factor to increase intervention effect. This information may help judge the concern given to possible confounding. Were the intervention arms comparable at baseline for the subgroup of interest? For example, if the subgroup of interest is sex, the systematic reviewer should try to confirm that males in the intervention group were comparable to males in the control group. Similarly, females in the intervention group should be comparable to females in the control group. If the stratified intervention arms are not comparable at baseline, secondary factors affecting comparability could be confounding study variables [54]. Was the subgroup variable a characteristic specified at baseline (in contrast with after randomization)? This ensures that the benefits of randomization are maintained throughout the duration of the study, and reduces the possibility of confounding [8]. The credibility of subgroup hypotheses based on *post*-randomization characteristics can be severely compromised, since any apparent difference in intervention effect could potentially be explained by the intervention itself or different prognostic characteristics in subgroups that emerge after randomization [57]. Analyses based on characteristics that emerge during follow-up violate the principles of randomization and are less valid [26]. Was the subgroup variable a stratification factor at randomization? Randomization stratified for a priori subpopulations ensures comparable distribution of other characteristics, including potential confounding factors between subgroups on this factor [24, 54]. Stratified randomization ensures there is a separate randomization procedure within each subset of participants.
Likelihood of inadequate POWER to detect subgroup differences	Was the trial powered to detect subgroup differences? If important subgroup-intervention effect interactions are anticipated, trials should be powered to detect them reliably [18, 54]. If a trial is underpowered for the main outcomes of interest, it is almost never adequately powered for a subgroup analysis. If a study *did* detect a difference in subgroup effect, then this domain would be assessed as very unlikely (i.e., that power was inadequate) because the power calculation, which was based on assumptions such as an estimate of the difference that *might* exist, is no longer very important after a significant difference has been revealed. If a study does *not* detect a difference, then it is very relevant to assess whether or not the study was underpowered. To inform judgments made about the evidence, the Grading of Recommendations, Assessment, Development and Evaluation (GRADE) Working Group suggests that systematic reviewers consider the optimal information size (OIS) threshold as an additional criterion for adequate precision. OIS is reached if the total number of patients included in a systematic review is the same or more than the number of patients generated by a conventional sample size calculation for a single adequately powered trial [58]. Another potential application of the OIS criterion could be to indicate potential power issues in important subgroup analyses.

Table 8 outlines specific questions about spurious findings, confounding, and power limitations to assist reviewers in their credibility assessment of subgroup-specific analyses for a priori subpopulations. Based on responses to the questions outlined in Table 8 and whether observed subgroup effects are biologically plausible and consistent with evidence from related studies [8, 24], systematic reviewers can assess the credibility of each subgroup analysis reported by the study by judging each of the three domains (spurious, confounding, power) as very likely, somewhat likely, unlikely, or unclear—usually due to inadequate reporting (Table 7). The spurious effects domain would also include a “not applicable” option when indicating credibility assessment for situations when a study does not detect a difference in subgroup effect.

Reviewers can summarize their study-level subgroup analysis-specific credibility assessment with an overall rating (e.g., low, medium, high, or uncertain) that incorporates the results of each relevant domain (Table 7). This overall subgroup analysis credibility rating represents a summary judgment as to the credibility of the subgroup-specific analyses conducted in each study of interest and is therefore taken into consideration within the larger context of the study’s internal validity (risk of bias) from the critical appraisal process.

Finally, studies that only enrolled an a priori subpopulation (e.g., 100% female) can be assessed for quality as part of the routine quality rating process for all studies. Ancillary reports from included studies reporting relevant subgroup analyses can also be assessed for credibility using Tables 7 and 8.

### Phase III: data analysis and synthesis

#### Investigating potential sources of heterogeneity at the body of evidence level

During the data analysis and synthesis phase of systematic reviews, reviewers summarize the body of evidence, appropriately considering differences between studies in terms of clinical, methodological, and statistical heterogeneity (Table 2). Guided by a priori considerations, reviewers can supplement their systematic consideration of the similarities and differences across trials in the body of evidence using the PICOTS (population, intervention, comparator, outcome, timing, and study design) rubric (Table 9) [26]. Population factors may drive important clinical heterogeneity based on issues such as baseline study group risk for intervention-related benefits or harms. In contrast, between-study differences in the study design or conduct can represent methodological heterogeneity that is not clinically meaningful, while intervention and comparator differences may or may not be clinically relevant. The consistency and variability in the body of evidence may not be evident when abstracting data from individual studies, so reviewers should consider the consistency and variability in all factors across included studies at this point in the process.

**Table 9:** Framework for reviewing factors influencing heterogeneity across included studies

1. Population	2. Intervention	3. Comparator	4. Outcomes	5. Timing and tools	6. Study design and conduct
Heterogeneity factors for each major domain driving heterogeneity					
- Baseline risk for primary outcome (without intervention) as well as for intervention-related harms - Other main population differences hypothesized to drive differences in intervention effects	- Differences in the approach, intensity, modalities, or components of interventions that could drive differences in intervention effects	- Components of comparison condition that might influence the size/direction of intervention effects	- Comparability of inpatient outcomes across studies that might influence intervention effects	- Appropriateness and comparability of outcome assessment timing considering hypothesized intervention effects and natural history	- Variability in design and conduct of studies within a body of evidence
Potential categories of variable approaches by individual studies					
- Risk based (low, average, high, unclear, mixed) - Other selected (age, race/ethnicity, sex, education, socioeconomic status)	- Approach (generic, targeted, tailored) - Intensity/dose (hours, duration, staff) - Modalities (simple, multiple) - Components (single, co-interventions)	- Placebo - Usual care - Active/alternative treatment - Incremental effect (intervention and comparator only, vary by one or minimal components)	- Type of outcome (primary, secondary, incidental) - Number and type of beneficial outcomes (one main, multiple, composite) - Number and type of harmful outcomes (one main, multiple, composite) - Validity of outcome measurement	- Appropriateness (measured or timed after intervention ended, delayed measurement at meaningful timeframes - Comparability (consistent timeframe between studies, variable timeframe for study)	- Quality rating (good, fair, poor) - Risk of bias (lack of allocation concealment, lack of blinded outcome assessment, inappropriate randomization)

When looking at the body of evidence, reviewers should consider variability across studies in the baseline population risk for the primary outcome for which the intervention is intended since this is one of the primary drivers of heterogeneity, along with variable risk for intervention-related harms or presence of competing risks. Even when an intervention has the same relative effects across subpopulations, the absolute benefits will vary, producing much larger beneficial effects in those at higher baseline risk. Thus, understanding the range of baseline risks represented across the body of evidence can be important to interpret findings, whether represented by absolute or relative effect measures.

Observed variation in population risk (as sometimes approximated by control group event rates) across studies may reflect not only different patient populations with variability in baseline risk among selected groups but also other factors such as length of study follow-up [27]. The control group event rate can also be viewed as a study-level proxy for disease severity, concomitant treatments, and follow-up duration [28]. Visual inspection of scatter plots or inspection of data in a spreadsheet to consider the extent of variability in baseline population risk (or any factor) across the body of evidence can be a useful initial assessment of heterogeneity [29].

**Fig. 2:**
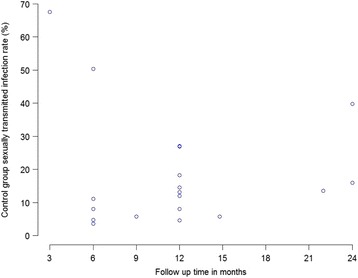
Scatter plot of control group event rates for the primary outcome of sexually transmitted infections (by longest time points for each study) [30]

For example, Fig. 2 shows a scatter plot of control group event rates for the primary outcome of sexually transmitted infections by follow-up time [30]. The broad range of control group rates across 3 to 24 months of follow-up suggests potential population differences in baseline risk of sexually transmitted infection. Scatter plots may also be used to consider the extent of variability in intervention-related risks (control group event rates for harms by follow-up time) or the relationship between baseline risk and absolute benefit (intervention group event rates by control group event rates). Reviewers may also use forest plots to investigate heterogeneity of intervention effects, stratified by population type or other important variables for appropriate time points.

Systematic reviewers consider whether intervention effects are relatively homogeneous or appear to show variable effects on primary outcomes, including benefits and harms. This examination includes, but is not limited to, using appropriate statistical methods to examine the consistency and precision of the overall findings. The decision to mathematically combine data depends on critical judgment [31]. A meta-analysis should only be conducted when a group of studies is considered homogeneous enough in terms of population, interventions, and outcomes that combining would produce a meaningful summary [7]. The underlying biology should suggest that it is plausible that the magnitude of effect on the key outcomes should be more or less the same across the range of patients and interventions [9, 26]. If meta-analyses are deemed appropriate given the body of evidence, systematic reviewers should determine appropriate statistical methods for meta-analyses and explorations of heterogeneity by first consulting respected scientific literature and statisticians when necessary. A detailed discussion of these methods is beyond the scope of this paper.

**Fig. 3:**
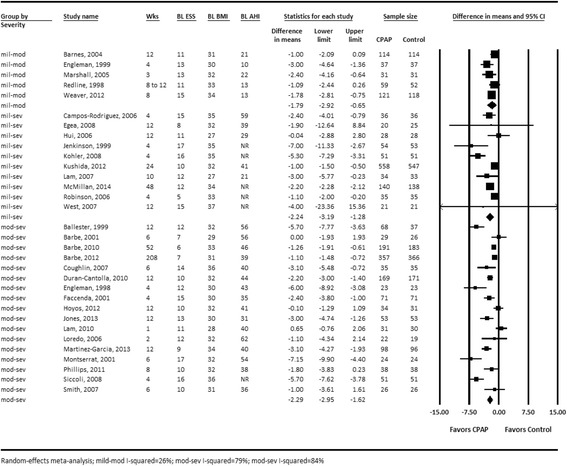
Forest plot of the effect of continuous positive airway pressure (CPAP) on sleepiness (by obstructive sleep apnea (OSA) severity at baseline) [6]

Systematic reviewers should formally assess potential heterogeneity using common statistical approaches to detect and quantify the degree of heterogeneity (i.e., Cochran’s Q test, *I* ^2^ index) [32, 33]. If reviewers determine that statistical heterogeneity is present, further exploration is needed to investigate the potential sources of heterogeneity. Even when statistical heterogeneity is not present, a priori factors may still need to be explored [19], particularly since lack of statistical heterogeneity does not confirm lack of either clinical or methodological heterogeneity and statistical tests are generally considered to be underpowered to detect differences in subgroup effects [34]. Common approaches for such investigations include stratified meta-analyses, sensitivity analyses, and meta-regression [35]. For example, Fig. 3 is a stratified meta-analysis that provides pooled estimates for subgroups defined by sleep apnea severity at baseline for the effect of continuous positive airway pressure (CPAP) on sleepiness as measured by the Epworth Sleepiness Scale [6]. This type of approach provides information on the degree to which effect sizes differ between groups of studies and also shows whether a substantial portion of the statistical heterogeneity was caused by combining sets of studies into one meta-analysis. When conducting these types of analyses, reviewers must consider potential limitations, such as confounding, inadequate variability, ecological fallacy, and power [8, 11, 17]. Reviewers may also employ graphical methods that more broadly identify potential sources of heterogeneity, being careful to distinguish a priori from post hoc factors [36].

If meta-analyses are not appropriate given the body of evidence, reviewers should provide a narrative synthesis of results, stratified by potential sources of heterogeneity identified a priori. Systematic reviewers should describe the individual study results in the context of the apparent heterogeneity (or lack thereof) in the evidence. In the absence of formal quantitative synthesis, forest plots may still be used to display intervention effects, stratified by population type or other important variables for appropriate time points, to enhance communication.

### Summarizing findings at the subpopulation level

During this phase, reviewers also consider the findings from relevant subgroup data abstracted during phase II for each subpopulation. This requires summarizing whether subgroup-specific findings were available from individual studies and how credible they were, as well as their overall coherence across studies. Considered together with results from examining the body of evidence for important heterogeneity of intervention effects, these findings will carry forward to inform judgments by the guideline developer about the possible need for subpopulation-specific clinical practice recommendations.

In order to summarize subgroup findings for examination, systematic reviewers can complete Table 10 for each a priori subpopulation as appropriate after reviewing the credibility and availability of subgroup analyses abstracted across all of the included studies during phase II. This can be most useful when there are a sufficient number of studies reporting subgroup-specific or related analyses for an a priori subpopulation of interest (e.g., age-, sex-, or race-specific). If there are few subgroup analyses reported, text descriptions will usually suffice. If there are extensive subgroup analyses reported, reviewers may want to limit the analyses abstracted to those with at least a moderate overall credibility rating. Additionally, if reviewers have noted a consistently reported set of subgroup analyses for an important subpopulation, or studies targeting that same subpopulation, but the subpopulation was not identified a priori, it may be appropriate to summarize this information post hoc in text or in a summary table, with clear labeling that these represent exploratory findings.

**Table 10:** Subpopulation-specific summary table with example

Study name (quality rating)	Subgroup analysis credibility rating (*from phase II*)	(A) What is the definition of the subgroup in this study?	(B) What are the results of the subgroup-specific interaction test?	(C) What are the results of subgroup-specific analyses for this subpopulation in this study?	(D) What are the results of other subgroup-relevant analyses for this subpopulation in this study?
Study A (enter quality rating)	Enter subgroup analysis credibility rating from phase II	Clearly define the subgroup (or subpopulation) as described in the study (e.g., ages 65 years and older).	Abstract results of formal tests for interaction, and indicate the presence or absence of statistical significance (i.e., not significant (NS), significant (S), or not reported (NR)) and all available *p* values. Note: The correct analysis is not to test the significance of the intervention effect in one subgroup or another, but whether the effect differs significantly between subgroups [54, 59].	Abstract results of subgroup-specific stratified analyses conducted in the study (e.g., *p* values and intervention-effect measures of association [odds ratios, relative risks, mean changes] reported by subgroup) [53]. Enter the intervention effect with 95% confidence intervals for the main average and subgroup-specific analyses. Report results of subgroup analyses as absolute and relative risk reductions. Absolute risk reduction estimates give the probability an individual will benefit from an intervention [60]. Note: Estimates can be generated for patients with differing baseline risks that represent types of patients seen in clinical practice by multiplying baseline risk by a pooled homogeneous relative risk estimate [61, 62].	Abstract numerical results and statistical tests of other types of relevant subgroup analyses (e.g., findings with and without subgroup-adjustment in a logistic regression model, multivariable analyses predicting outcomes including subgroup variables).
Women’s Health Study Ridker, 2005 [63] (Good)	Moderate	Age groups: 45–55 years 55–64 years ≥65 years	Interaction test for outcome of total myocardial infarction (MI): *p* = 0.03 (S)	Relative risk reduction (95% CI) for total MI: Main average effect: 1.02 (0.84 to 1.25), *p* = 0.83 45–55 years: 1.23 (0.87 to 1.75), *p* = 0.25 55–64 years: 1.17 (0.86 to 1.59), *p* = 0.32 ≥65 years: 0.66 (0.44 to 0.97), *p* = 0.04 Absolute risk reduction (95% CI) for total MI (calculated): Main average effect: 0.000 (−0.002 to 0.002) 45–55 years: −0.001 (−0.003 to 0.001) 55–64 years: −0.002 (−0.006 to 0.002) ≥65 years: 0.010 (0.001 to 0.020) *p* = 0.04	NA

The synthesis of subpopulation-specific findings considers the (1) volume and credibility of subgroup analyses, (2) overall coherence of findings, and (3) limitations. The volume and credibility of subgroup analyses will depend on the total number of participants represented and the number of studies reporting subpopulation-specific results out of the total number of included studies, as well as the quality of the evidence, judged by threats to credibility of available subgroup-specific study results and availability of within-study versus between-study subpopulation comparisons. The overall coherence of findings can be assessed by reviewing the consistency of subgroup/subpopulation findings across trials [26], the way subgroups are defined, credibility of subgroup analyses, comparability of studies focused on the specific subpopulations within the body of evidence in terms of PICOTS, number of studies reporting results for each subgroup by outcome, and comparison of within-study to between-study subgroup results. Finally, systematic reviewers should summarize the limitations of the evidence, including potential confounders in individual study subgroup analyses, potential confounders in the study designs, and gaps or deficiencies in the subpopulation-specific results.

**Fig. 4:**
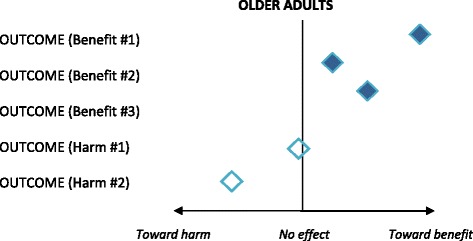
Example summary plot for relevant subpopulation-specific outcomes

Reviewers may create summary plots for outcomes of interest to facilitate considerations of net benefit. These should always include both benefits and harms. After transformation to allow statistically combined estimates to reflect the appropriate direction for a finding (i.e., toward benefit or toward harm), summary estimates can be reflected on a plot (Fig. 4).

### Phase IV: reporting and interpretation

#### Reporting

The value of a systematic review depends on the methods, findings, and clarity of reporting [37]. Transparency and consistency are keys to any systematic approach, with methods and the rationale for decisions and subjective judgments clearly articulated. As such, systematic reviewers should clearly communicate the approach taken in the review to assess heterogeneity, the types of data available, judgments about the presence or absence of important clinical heterogeneity, and appropriate limitations and caveats, as determined by thorough investigation of data at both the overall body of evidence and subpopulation levels. Findings must be sufficiently clear to inform judgments about the adequacy of the evidence base for specific subpopulations and appropriateness of general population versus subpopulation-specific clinical practice recommendations, as well as allow for incorporation into future research considerations. A structured approach to reporting facilitates interpretation as well as communication of data throughout this phase.

A list of elements to include when reporting patient subpopulation findings in a systematic review is displayed in Table 11. Authors should adhere to the PRISMA (Preferred Reporting Items for Systematic Reviews and Meta-Analyses) Statement [37] when reporting a systematic review [19]. We have added elements specific to subpopulations and heterogeneity of intervention effects to augment this suggested reporting approach.

**Table 11:** Summary of elements to include when reporting a systematic review

Report section	Reporting elements
Abstract	- Report valid, a priori subgroup or subpopulation findings in the structured abstract. - Report non-valid or insufficient evidence if it is a critical clinical or policy issue.
Introduction	- Summarize the rationale for specific subpopulation considerations, including disease burden and potential differences in expected harms or benefits from the clinical preventive service, based on previous research [18].
Methods	- Briefly summarize the approach used to identify important subpopulation considerations in the review (e.g., literature searches, clinical and content expert consultation, and public comments). - Identify the a priori subpopulations the review addressed and the approaches taken for locating these data. - Clearly report how subgroups were defined (e.g., by categorical predictors or continuous risk scores) [18]. - Describe methods for abstracting subgroup and related analyses and any quality control processes, such as dual reviewing extracted data from primary studies [18]. - Describe methods for assessing the credibility of subgroup analyses related to a priori subpopulations at the study level and for focusing the report on clinically meaningful subpopulation results in the body of evidence. - Report methods used to explore heterogeneity of intervention effects [18]. Describe methods for additional analyses (e.g., sensitivity or subgroup analyses, meta-regression), if conducted, indicating those that were specified a priori [37].
Results	- Summarize qualitative heterogeneity of body of evidence at methodological and clinical levels. - Report all proposed and actual investigations of clinical heterogeneity differentiating prespecified and post hoc, including all subgroups and outcomes analyzed [18, 19, 61]. - Summarize the frequency of subgroup analyses for a priori subgroups, the credibility of available subgroup analyses, and overall coherence of findings. - Report whether within-study results showed statistical evidence of effect modification by baseline subpopulation or other important characteristics across studies [64]. - Report results of meta-regression or other pooled subpopulation analyses if conducted. Report judgments or findings of clinical, methodological, or statistical heterogeneity. - Summarize results of subgroup analyses as absolute risk reductions and relative risk reductions. - Report any subpopulation differences in rates of serious harms. Report any other factors strongly associated with these harms [65]. - Any reported results from post hoc subgroups or subpopulations should be labeled exploratory.
Discussion	Summary of evidence - Summarize the main findings for the overall body of evidence and subpopulations of interest [37]. - Report on all a priori subgroups, whether reporting on the absence of data to evaluate, an absence of detected effect modification (for relative or absolute measures), or detectable effect modification (on which scale), and its clinical significance. - Clearly report and distinguish between evidence of no effect, uncertain or incomplete evidence, or lack of evidence. - Clearly state when evidence may warrant separate considerations of net benefit in subpopulations. - Clearly indicate if caution is warranted in applying the average effect for some types of patients, even if evidence is unavailable or limited. Limitations - Summarize limitations of subgroup and subpopulation findings at the study, outcome, and review levels based on gaps in the evidence. Future research - Reference important exploratory findings from post hoc subgroups. - Provide recommendations on how future research could proceed or build upon results vis-à-vis important subpopulations. Conclusions - Provide a general interpretation of any a priori subpopulation findings in the context of other evidence.

The most informative approach to summarizing the results of subgroup analyses may be to use the review’s overall summary (or strength) of evidence table and stratify the body of evidence by subpopulation within the appropriate key question(s), especially when the subgroup data may be the basis for considering a subpopulation-specific recommendation or clinical consideration. Using the summary of evidence table allows reviewers to consistently and transparently present summary evaluations of each evidence domain (e.g., consistency, precision, reporting bias, body of evidence limitations, strength of evidence, applicability) for important subpopulations [38]. The summary of evidence table can show how the subpopulation-specific information fits within the overall body of evidence and organization of the topic. The level of stratification used for subpopulations depends on the way a topic is conceptualized; for example, some topics may be stratified by intervention type first, with the subpopulation as the second order of stratification. For other topics, subpopulation evidence may only vary for specific domains so would only be presented for a particular domain (e.g., precision).

For example, a review of screening for obstructive sleep apnea (OSA) assessed whether benefits of treatment with CPAP differ for subpopulations defined by OSA severity (among other subpopulation questions considered) [39]. The review conducted subgroup meta-analyses by OSA severity categories. One approach to presenting those findings in an overall summary of evidence table would be to enter data in separate rows for the full sample and for each of the subpopulations, such that treatment with CPAP has a row for overall findings (for the full population) and also has rows for each subpopulation (e.g., mild OSA, moderate OSA, and severe OSA). Such an approach might be most useful when there are significant differences for multiple evidence domains between the overall population and subpopulations (such that reviewers want to highlight the details of similarities and differences). The main conclusions of credibility assessments from phase II would contribute to subpopulation domain entries for quality/risk of bias and body of evidence limitations. Alternatively, depending on how the topic was conceptualized and the specific review findings, the results for subpopulations might be highlighted (1) only in the applicability domain or (2) within a single row dedicated to the effects of treatment with CPAP that first shows effects for the overall population for each domain and then (below the overall findings) describes any differences for subpopulations that were identified and the credibility of those findings.

### Interpretation

Systematic reviewers must consider how to interpret the overall credibility of subgroup analyses reported by the studies included in a review. Considerations for judging the credibility of subpopulation findings at the body of evidence level include:

- Are the subgroup analyses upon which any subpopulation analyses are based credible and consistent across studies and outcomes?
- Do subpopulation findings avoid ecologic fallacy (i.e., are they based upon meta-regression involving only appropriate study-level variables or using appropriate individual participant data meta-analyses for patient-level variables)?
- Were the subpopulation analyses in the systematic review specified a priori in a specific hypothesized direction?
- Was the total number of subpopulation investigations in the systematic review limited to a small number?
- Does statistical analysis suggest chance is an unlikely basis for subpopulation differences?
- Are subpopulation findings supported by within-study findings rather than, or in addition to, between-study comparisons?
- To what extent are subpopulation findings biologically plausible? [10]

Table 12 provides caveats to assist systematic reviewers in their interpretation and understanding of the available subpopulation-specific data. The caveats stress the importance of caution in the interpretation of subgroup analyses due to the risk of false positive or false negative subgroup effects. Guidelines based on spurious subgroup analyses could result in subpopulations of patients receiving inappropriate treatment or being denied beneficial treatment. When data are not definitive, the average intervention effect is considered the best estimate [40, 41]. Pilot testing confirmed that this phase IV guidance provides useful caveats to explain the limitations of subpopulation findings and ensures that clear reporting of subpopulation evidence is not neglected.

**Table 12:** Caveats for interpreting and understanding subpopulation-specific data

Availability of subpopulation-specific data	Caveats
Presence of subpopulation differences in intervention effects	- When interpreting the presence of subgroup or subpopulation-specific findings, recall that evidence is usually observational [7]. Consider methodological heterogeneity, confounding and other sources of bias (e.g., publication, misclassification), magnitude and direction of effect and confidence intervals, and plausibility of causal relationships. Confounding can lead to spurious or misleading subgroup results, particularly when subgroup factors are correlated [61]. - When interpreting reported subgroup effects, beware of false positive effects. If multiple subgroup analyses are conducted, the probability of a false positive finding can be high [55]. Results are more likely to be real if they are based on a priori analyses because these have prior evidence supporting them. - When claiming an intervention effect in a subgroup, consider whether appropriate methods (e.g., *p* value adjustment, false discovery rates, Bayesian shrinkage estimates, adjusted confidence intervals, or internal or external validation methods) were used to account for the number of contrasts examined [18].
Absence of subpopulation differences in intervention effects	- Subgroup analyses are typically underpowered, thus the risk of false negatives is even higher. One should be aware of the remaining possibility of false negatives in the absence of relative intervention effect differences [59]. - Lack of relative intervention effect differences between subgroups may still result in clinically important variations in absolute benefit due to the impact of differences in baseline risk on absolute intervention effect. - Lack of difference between subgroups defined on single factors (e.g., age, race/ethnicity) is not sufficient reasoning that subpopulation differences do not exist. Subgroups defined through multivariable risk prediction tools are more likely to be clinically applicable and robust, particularly with larger studies. If a body of evidence has similar multivariable subgroup definitions within studies, pooling can increase power [66]. - Even without heterogeneity of intervention effects, not everyone who receives a “proven” intervention will benefit. (For an intervention with a constant 25% relative risk reduction, one-quarter of expected events will be averted, but 75% of events will still occur despite intervention) [67]. Reminding readers of this fact and emphasizing absolute effects within overall event rates is informative. Further, this approach can help clarify why even modest risk of serious harms may, in the end, exert a strong impact on net benefit calculations for the population as well as for individuals [66]. - When data are not definitive and overall benefits are modest, or overall benefits are moderate but intervention is costly, retaining the possibility of heterogeneity of intervention effects in the absence of evidence may be warranted. Consideration of individualized or targeted intervention approaches may still be applicable for future studies. - In the absence of compelling evidence, the best estimate is the average intervention effect [40].
Overall	- If meta-analyses were conducted, reviewers should consider possible explanations of variations between clinical and statistical heterogeneity. - Caution is warranted for definitive subgroup conclusions in the absence of patient-level meta-analysis or valid study-level methods and replication (or pooling) of within-study subgroup-specific findings across trials [54]. - Intervention-related risks are substantial (at least for some) and factors that appear to predict increased risk for serious harms can be related to subpopulations. When serious harms are a key issue, consider looking for validated risk prediction tools for serious harms to assist in net-benefit considerations, whether or not reviewed data support subgroup differences [40]. - Data to robustly support subgroup and heterogeneity of intervention evaluations are generally not available given the current state of clinical trial reporting [68]. As a result, predicting individual effects occurs less often, even though it is an area of growing interest as the field of precision medicine develops [18, 69]. Recent recommendations may improve the assessment and reporting of heterogeneity in clinical trials going forward [59].

## Conclusions

In our work conducting systematic reviews for the USPSTF, we increasingly face the need to provide information on how treatment effects differ for some groups of patients to inform decisions about the appropriateness of subpopulation-specific clinical practice recommendations. Therefore, among a set of reviewers working across Evidence-based Practice Centers—and in conjunction with the USPSTF Subpopulation Workgroup—we developed the guidance described in this paper for addressing subpopulation considerations in systematic reviews. We would welcome engaging in an international consortium effort to develop consensus methods as a next step.

Rigor and comprehensiveness are important to good systematic review methods, but reviewers have to work within the time and resource constraints imposed by those commissioning the review and the guideline developers or others who will use the results. Therefore, it is essential to consider whether the value of adding the subpopulation processes detailed here justifies the additional time and effort expended. The additional work necessary to define subpopulations of interest a priori during initial planning can actually reduce the time and effort spent in later stages of the review by limiting subpopulation examinations to those of most significance to a particular topic. For some topics, early investigations of subpopulations during topic scoping may result in a conclusion that further consideration of subpopulations is not warranted. Systematic investigation of potential heterogeneity in a body of evidence, along with quantitative and narrative analysis and synthesis of subgroup data, represents considerable time and effort and adds a substantial amount of work to the overall review process. The net value of this process is therefore contingent on the effectiveness of earlier phases of the review in identifying the most important subpopulations for a topic and determining the availability of credible subgroup data.

Understanding how treatment benefits and harms differ across patient populations is necessary for optimal patient care and is increasingly focused on through “precision medicine”; therefore, methods to incorporate subpopulation considerations, including credible subgroup analyses, into systematic reviews and clinical practice guidelines are increasingly important. Our proposed approach is intended to allow systematic reviewers to more robustly and routinely provide information about which subpopulations differ enough in the likelihood of benefits (and/or risk of harms) from a preventive intervention that they may warrant different clinical preventive recommendations. Gaps in the evidence on important subpopulations identified by applying this process in systematic reviews can also suggest future research needs. Although the processes we describe here were developed for systematic reviews to support recommendations made by the USPSTF, they are likely generalizable to systematic reviews in other clinical and policy contexts with minimal modification. We anticipate that this approach will undergo further refinement with additional use in reviews for the USPSTF and may require revisions to provide utility to the producers and users of systematic reviews beyond the context of the USPSTF and to broaden its application to reviews of evidence from non-randomized studies.

## Abbreviations

AAFP: American Academy of Family Physicians
AAP: American Academy of Pediatrics
ACOG: American Congress of Obstetricians and Gynecologists
ACP: American College of Physicians
AHRQ: Agency for Healthcare Research and Quality
CI: Confidence interval
CPAP: Continuous positive airway pressure
CPSTF: Community Preventive Services Task Force
CTFPHC: Canadian Task Force on Preventive Health Care
CVD: Cardiovascular disease
EPC: Evidence-based Practice Center
GRADE: Grading of Recommendations Assessment, Development and Evaluation
IOM: Institute of Medicine
MI: Myocardial infarction
NICE: National Institute for Health and Care Excellence
NR: Not reported
NS: Not significant
OIS: Optimal information size
OSA: Obstructive sleep apnea
PAD: Peripheral artery disease
PICOTS: Population, intervention, comparator, outcome, timing, and study design
PRISMA: Preferred reporting items for systematic reviews and meta-analyses
RCT: Randomized clinical trial
S: Significant
USPSTF: U.S. Preventive Services Task Force

## References

[1] Varadhan R, Segal JB, Boyd CM, Wu AW, Weiss CO (2013). A framework for the analysis of heterogeneity of treatment effect in patient-centered outcomes research. J Clin Epidemiol.

[2] West SL, Gartlehner G, Mansfield AJ, Poole C, Tant E, Lenfestey N (2010). Comparative Effectiveness Review Methods: Clinical Heterogeneity.

[3] Gagnier JJ, Moher D, Boon H, Beyene J, Bombardier C (2012). Investigating clinical heterogeneity in systematic reviews: a methodologic review of guidance in the literature. BMC Med Res Methodol.

[4] Chou R, Dana T, Blazina I, Daeges M, Bougatsos C, Grusing S (2015). Statins for prevention of cardiovascular disease in adults: systematic review for the U.S. Preventive Services Task Force.

[5] Guirguis-Blake JM, Evans CV, Senger CA, Rowland MG, O'Connor EA, Whitlock EP (2015). Aspirin for the primary prevention of cardiovascular events: a systematic evidence review for the U.S. Preventive Services Task Force.

[6] Jonas DE, Amick HR, Feltner C, Palmieri Weber R, Arvanitis M, Stine A (2016). Screening for obstructive sleep apnea in adults: an evidence review for the U.S. Preventive Services Task Force.

[7] Higgins JPT, Green S (editors). Cochrane Handbook for Systematic Reviews of Interventions Version 5.1.0 [updated March 2011]. The Cochrane Collaboration; 2011. Available from www.handbook.cochrane.org.

[8] Guyatt GH, Oxman AD, Kunz R, Woodcock J, Brozek J, Helfand M (2011). GRADE guidelines: 7. Rating the quality of evidence—inconsistency. J Clin Epidemiol.

[9] Guyatt GH, Oxman AD, Kunz R, Atkins D, Brozek J, Vist G (2011). GRADE guidelines: 2. Framing the question and deciding on important outcomes. J Clin Epidemiol.

[10] Moberg J, Alonso-Coello P, Oxman AD. GRADE Evidence to Decision (EtD) Frameworks Guidance. Version 1.1 [updated May 2015], The GRADE Working Group, 2015. Available from: https://ietd.epistemonikos.org/#/help/guidance.

[11] Cochrane Child Health. Methods for child health reviews. http://childhealth.cochrane.org/methods-child-health-reviews. Accessed 24 Aug 2016.

[12] Canadian Task Force on Preventive Health Care. Procedure manual. Canadian Task Force on Preventive Health Care. 2014. http://canadiantaskforce.ca/wp-content/uploads/2016/12/procedural-manual-en_2014_Archived.pdf. Accessed 11 Nov 2015.

[13] National Institute for Health and Care Excellence. The Guidelines Manual. National Institute for Health and Care Excellence. 2012. http://www.nice.org.uk/article/PMG6/chapter/1%20Introduction. Accessed 11 Nov 2015.

[14] IOM (Institute of Medicine) (2011). Finding what works in health care: standards for systematic reviews.

[15] Welch V, Petticrew M, Petkovic J, Moher D, Waters E, White H (2016). Extending the PRISMA statement to equity-focused systematic reviews (PRISMA-E 2012): explanation and elaboration. J Clin Epidemiol.

[16] National Institute for Health and Clinical Excellence. Positively equal: a guide to addressing equality issues in developing NICE clinical guidelines. 2nd ed. 2012.

[17] Bibbins-Domingo K, Whitlock E, Wolff T, Ngo-Metzger Q, Phillips WR, Davidson KW, et al. Developing recommendations for evidence-based clinical preventive services for diverse populations: methods of the U.S. Preventive Services Task Force. Annals of Internal Medicine. 2017. In press. 10.7326/M16-2656 28265649

[18] PCORI (2013). (Patient-Centered Outcomes Research Institute) Methodology Committee. The PCORI Methodology Report.

[19] Gagnier JJ, Morgenstern H, Altman DG, Berlin J, Chang S, McCulloch P (2013). Consensus-based recommendations for investigating clinical heterogeneity in systematic reviews. BMC Med Res Methodol.

[20] The Grade Working Group. GRADE Guidelines—best practices using the GRADE framework. In: The GRADE Working Group. The GRADE Working Group. 2014. http://www.gradeworkinggroup.org/. Accessed 11 Nov 2015.

[21] U.S. Preventive Services Task Force (2015). U.S. Preventive Services Task Force Procedure Manual.

[22] Effective Health Care Program (2011). Methods guide for effectiveness and comparative effectiveness reviews.

[23] Altman DG, Schulz KF, Moher D, Egger M, Davidoff F, Elbourne D (2001). The revised CONSORT statement for reporting randomized trials: explanation and elaboration. Ann Intern Med.

[24] Sun X, Briel M, Busse JW, You JJ, Akl EA, Mejza F (2012). Credibility of claims of subgroup effects in randomised controlled trials: systematic review. BMJ.

[25] Petticrew M, Tugwell P, Kristjansson E, Oliver S, Ueffing E, Welch V (2012). Damned if you do, damned if you don’t: subgroup analysis and equity. J Epidemiol Community Health.

[26] Sun X, Ioannidis JP, Agoritsas T, Alba AC, Guyatt G (2014). How to use a subgroup analysis: users’ guide to the medical literature. JAMA.

[27] Schmid CH, Lau J, McIntosh MW, Cappelleri JC (1998). An empirical study of the effect of the control rate as a predictor of treatment efficacy in meta-analysis of clinical trials. Stat Med.

[28] Lau J, Terrin N, Fu R (2013). Expanded guidance on selected quantitative synthesis topics. Methods Guide for Effectiveness and Comparative Effectiveness Reviews.

[29] Fu R, Gartlehner G, Grant M, Shamliyan T, Sedrakyan A, Wilt TJ (2011). Conducting quantitative synthesis when comparing medical interventions: AHRQ and the Effective Health Care Program. J Clin Epidemiol.

[30] O'Connor EA, Lin J, Burda BU, Henderson JT, Walsh ES, Whitlock EP (2014). Behavioral sexual risk reduction counseling in primary care to prevent sexually transmitted infections: an updated systematic evidence review for the U.S. Preventive Services Task Force.

[31] Cornell JE, Mulrow CD, Localio R, Stack CB, Meibohm AR, Guallar E (2014). Random-effects meta-analysis of inconsistent effects: a time for change. Ann Intern Med.

[32] Cochran WG (1954). The combination of estimates from different experiments. Biometrics.

[33] Higgins JP, Thompson SG (2002). Quantifying heterogeneity in a meta-analysis. Stat Med.

[34] Velentgas P, Dreyer NA, Nourjah P, Smith SR, Torchia MM (2013). Developing a protocol for observational comparative effectiveness research: a user’s guide.

[35] Gartlehner G, West SL, Mansfield AJ, Poole C, Tant E, Lux LJ (2012). Clinical heterogeneity in systematic reviews and health technology assessments: synthesis of guidance documents and the literature. Int J Technol Assess Health Care.

[36] Baujat B, Mahe C, Pignon JP, Hill C (2002). A graphical method for exploring heterogeneity in meta-analyses: application to a meta-analysis of 65 trials. Stat Med.

[37] Moher D, Liberati A, Tetzlaff J, Altman DG (2009). Preferred reporting items for systematic reviews and meta-analyses: the PRISMA statement. Ann Intern Med.

[38] Berkman ND, Lohr KN, Ansari M, McDonagh M, Balk E, Whitlock EP (2013). Grading the strength of a body of evidence when assessing health care interventions for the effective health care program of the agency for healthcare research and quality: an update.

[39] U.S. Preventive Services Task Force (2014). Final research plan: obstructive sleep apnea in adults: screening.

[40] Scott IA, Guyatt GH (2010). Cautionary tales in the interpretation of clinical studies involving older persons. Arch Intern Med.

[41] Yusuf S, Wittes J, Probstfield J, Tyroler HA (1991). Analysis and interpretation of treatment effects in subgroups of patients in randomized clinical trials. Jama.

[42] American Academy of Family Physicians. Developing clinical practice guidelines. American Academy of Family Physicians, Leawood, KS. http://www.aafp.org/patient-care/clinical-recommendations/practice-guidelines.html. Accessed 11 Nov 2015.

[43] American Academy of Pediatrics. Clinical practice guidelines and policy implementation. American Academy of Pediatrics, Elk Grove Village, IL. https://www.aap.org/en-us/professional-resources/quality-improvement/Pages/Guidelines-and-Policy-Development.aspx. Accessed 11 Nov 2015.

[44] American Congress of Obstetricians and Gynecologists. Resources & publications. American Congress of Obstetricians and Gynecologists, Washington DC. http://www.acog.org/Resources_And_Publications. Accessed 11 Nov 2015.

[45] American College of Physicians. Guideline process: how does ACP develop clinical recommendations? American College of Physicians,. http://www.acponline.org/clinical_information/guidelines/guidelines/process.htm. Accessed 11 Nov 2015.

[46] Qaseem A, Snow V, Owens DK, Shekelle P (2010). The development of clinical practice guidelines and guidance statements of the American College of Physicians: summary of methods. Ann Intern Med.

[47] Community Preventive Services Task Force. Systematic review methods. Community Preventive Services Task Force,.http://www.thecommunityguide.org/about/methods.html. Accessed 11 Nov 2015.

[48] Briss PA, Zaza S, Pappaioanou M, Fielding J, Wright-De AL, Truman BI (2000). Developing an evidence-based guide to community preventive services—methods. The Task Force on Community Preventive Services. Am J Prev Med.

[49] The Cochrane Collaboration. Cochrane reviews. The Cochrane Collaboration,. http://www.cochrane.org/what-is-cochrane-evidence. Accessed 11 Nov 2015.

[50] IOM (Institute of Medicine). Clinical practice guidelines we can trust. The National Academies Press, Washington, DC. 2011. http://iom.nationalacademies.org/Reports/2011/Clinical-Practice-Guidelines-We-Can-Trust.aspx. Accessed 11 Nov 2015.

[51] Glasziou PP, Sanders SL (2002). Investigating causes of heterogeneity in systematic reviews. Stat Med.

[52] Guyatt G, Oxman AD, Akl EA, Kunz R, Vist G, Brozek J (2011). GRADE guidelines: 1. Introduction-GRADE evidence profiles and summary of findings tables. J Clin Epidemiol.

[53] Fernandez YG, Nguyen H, Duan N, Gabler NB, Kravitz RL (2010). Assessing heterogeneity of treatment effects: are authors misinterpreting their results?. Health Serv Res.

[54] Rothwell PM (2005). Treating individuals 2. Subgroup analysis in randomised controlled trials: importance, indications, and interpretation. Lancet.

[55] Lagakos SW (2006). The challenge of subgroup analyses—reporting without distorting. N Engl J Med.

[56] VanderWeele TJ, Knol MJ (2011). Interpretation of subgroup analyses in randomized trials: heterogeneity versus secondary interventions. Ann Intern Med.

[57] Sun X, Briel M, Walter SD, Guyatt GH (2010). Is a subgroup effect believable? Updating criteria to evaluate the credibility of subgroup analyses. BMJ.

[58] Guyatt GH, Oxman AD, Kunz R, Brozek J, Alonso-Coello P, Rind D (2011). GRADE guidelines: 6. Rating the quality of evidence—imprecision. J Clin Epidemiol.

[59] Kent DM, Rothwell PM, Ioannidis JP, Altman DG, Hayward RA (2010). Assessing and reporting heterogeneity in treatment effects in clinical trials: a proposal. Trials.

[60] Rothwell PM, Mehta Z, Howard SC, Gutnikov SA, Warlow CP (2005). Treating individuals 3: from subgroups to individuals: general principles and the example of carotid endarterectomy. Lancet.

[61] Egger M, Smith GD, Altman DG (2001). Systematic reviews in health care: meta-analysis in context.

[62] Glasziou PP, Irwig LM (1995). An evidence based approach to individualising treatment. BMJ.

[63] Ridker PM, Cook NR, Lee IM, Gordon D, Gaziano JM, Manson JE (2005). A randomized trial of low-dose aspirin in the primary prevention of cardiovascular disease in women. N Engl J Med.

[64] Thompson SG, Higgins JP (2005). Treating individuals 4: can meta-analysis help target interventions at individuals most likely to benefit?. Lancet.

[65] McAlister FA, Straus SE, Guyatt GH, Haynes RB (2000). Users’ guides to the medical literature: XX. Integrating research evidence with the care of the individual patient. Evidence-Based Medicine Working Group. JAMA.

[66] Atkins D, Chang SM, Gartlehner G, Buckley DI, Whitlock EP, Berliner E (2011). Assessing applicability when comparing medical interventions: AHRQ and the Effective Health Care Program. J Clin Epidemiol.

[67] Arias E (2011). United States life tables, 2007. Natl Vital Stat Rep.

[68] Gabler NB, Duan N, Liao D, Elmore JG, Ganiats TG, Kravitz RL (2009). Dealing with heterogeneity of treatment effects: is the literature up to the challenge?. Trials.

[69] The White House. The precision medicine initiative. Washington, DC. 2015. https://www.whitehouse.gov/precision-medicine. Accessed 11 Nov 2015.

